# Long-term surgical outcomes and prognostic factors of foveal detachment in pathologic myopia: based on the ATN classification

**DOI:** 10.1186/s12886-022-02391-1

**Published:** 2022-04-18

**Authors:** Jingyang Feng, Jiayi Yu, Qiuying Chen, Hao Zhou, Fenge Chen, Weijun Wang, Xun Xu, Ying Fan

**Affiliations:** 1 Department of Ophthalmology, Shanghai General Hospital (Shanghai First People’s Hospital), Shanghai Jiao Tong University School of Medicine, Shanghai, China; 2 National Clinical Research Center for Eye Diseases, Shanghai, China; 3 Shanghai Key Laboratory of Fundus Disease, Shanghai, China; 4 Shanghai Engineering Center for Visual Science and Photomedicine, Shanghai, China; 5 No.85 Wujin Road, 200080 Shanghai, China

**Keywords:** Foveal detachment, Vitrectomy, ATN classification, Myopic maculopathy, Prognostic factor

## Abstract

**Background:**

To investigate the long-term surgical outcomes and prognostic factors of foveal detachment (FD) in pathological myopia.

**Methods:**

This retrospective observational study included 59 patients with FD (61 eyes) who underwent pars plana vitrectomy at Shanghai General Hospital between June 2017 and July 2018 with follow-up for at least 24 months. Comprehensive ophthalmic examinations, including best-corrected visual acuity (BCVA) and swept-source optical coherence tomography, were assessed. Preoperative myopic maculopathy was evaluated according to the ATN classification.

**Results:**

FD completely resolved in 59 of 61 eyes (96.7%). Mean duration of retinal reattachment was 12.10 ± 8.10 months. Mean logMAR BCVA improved from 1.34 ± 0.52 to 0.83 ± 0.43 at 24 months postoperatively (*P* < 0.001). Secondary macular hole occurred in 8 eyes (13.1%) with a mean period of 3.4 ± 4.1 weeks after primary surgery. In regression analyses, baseline myopic atrophy maculopathy (MAM) (*B* = 0.213, *P* = 0.005) and vitreomacular traction (VMT) (*B* = 0.292, *P* = 0.007) were adverse prognostic factors for postoperative BCVA. A more severe MAM revealed a delay in retinal reattachment (*B* = 5.670, *P* = 0.002). FD eyes with VMT (OR = 1.309, *P* = 0.003) or outer lamellar macular hole (O-LMH) (OR = 1.369, *P* < 0.001) were risk factors for postoperative secondary macular hole.

**Conclusions:**

Vitrectomy was effective in the long-term for treating FD. Careful consideration is needed for those with VMT or O-LMH due to the high risk of secondary macular hole after vitrectomy. FD eyes with more severe MAM tended to have poorer postoperative BCVA and extended periods of retinal reattachment.

## Background

Maculopathy of pathologic myopia (PM) is an increasingly common cause of visual impairment and blindness worldwide, especially in East Asia [1]. It is estimated that approximately 4758 million people will have myopia by 2050, and up to one-fifth of them will have severe myopia, which foreshadows high morbidity of myopic maculopathy [2]. Foveal detachment (FD) without macular hole, a type of myopic tractional maculopathy (MTM), is an important sight-threatening complication of PM. The development of FD is thought to begin with myopic macular retinoschisis [3]. In the early stage of MTM, retinoschisis can be completely asymptomatic and may remain stable for a long period. However, it has been reported that in 34.5–72% of patients, macular retinoschisis advances to FD [4–6]. Once FD develops, further serious MTM, such as full-thickness macular holes and macular hole retinal detachment, proceed rapidly and ultimately lead to irreversible visual loss [7]. Thus, timely clinical intervention is needed to prevent the progression of FD.

There are two sets of forces that act on the retina during FD development: preretinal and subretinal factors [8]. The posterior vitreous cortex, epiretinal membrane, and internal limiting membrane (ILM) comprise preretinal factors that cause centrifugal and tangential forces to the macula. Vitrectomy combined with ILM peeling may release the tractions generated by preretinal factors. However, the surgical approach for FD is controversial, including the choice of tamponade materials, prognostic factors and long-term effectiveness. Several studies have investigated the vitrectomy approach for retinoschisis to block it evolution to FD or more severe maculopathy [9, 10]. However, the natural course of macular retinoschisis is variable. It is reported that 11.6% myopic retinoschisis experienced progression over 2 years of follow-up. Eyes with more extensive retinoschisis progressed more commonly (42.9%) than the eyes with less extensive retinoschisis (6.7%) [11]. Surgical intervention for all macular retinoschisis may increase the risk of overtreatment.

Current surgical research of MTM mainly concentrates on tractional lesions detected by optical coherence tomography. However, many MTM patients are complicated with other features of PM, such as atrophic or neovascular lesions, which may also influence surgical prognosis. Thus, we applied ATN classification for preoperative evaluation. This new grading system integrated three most important myopic maculopathy: atrophy (A), traction (T), and neovascularization (N) [12]. The confidence and reproducibility of the ATN system have been validated [13]. It may provide ophthalmologists with a more comprehensive and dependable approach for the preoperative evaluation of patients with FD.

Although vitrectomy has been advocated for treating MTM, the surgical management and long-term outcomes of FD still need further investigation. Considering the recent application of the ATN grading system, no research has applied it to explore the characteristics of atrophic or neovascular maculopathy in FD eyes and their relation to the surgical prognosis of FD. In this report, we followed up 61 FD eyes for at least 24 months to assess the baseline and long-term surgical outcomes in FD and to explore the prognostic factors for anatomic and functional recovery based on the ATN criteria.

## Methods

This was a retrospective observational study. The study was approved by the ethics committee of Shanghai General Hospital and adhered to the latest tenets of the Declaration of Helsinki. We reviewed medical records of 61 FD eyes in 59 PM patients who underwent 23-gauge pars plana vitrectomy with ILM peeling and silicone oil or sterilized air tamponade by a single surgeon (Y.F.) at Shanghai General Hospital between June 2017 and July 2018.

### Patient eligibility

Inclusion criteria: (1) patients with foveal detachment; (2) age more than 18; (3) refractive error greater than − 6.00 diopters and axial length longer than 26 mm; (4) postoperative follow-up longer than 24 months.

Exclusion criteria: (1) patients with full-thickness macular hole or any retinal holes before surgery; (2) a history of other retinal or choroidal disorders, such as uveitis, diabetic retinopathy, retinal vascular occlusion, exudative age-related macular degeneration; (3) severe cataract, glaucoma; (4) a history of previous vitreoretinal surgery; (5) eyes with low quality of ocular images; (6) uncontrolled systemic disease.

### Ophthalmic examinations

Patients’ data of comprehensive ophthalmic examinations were reviewed including refractive error (KR-8900; Topcon, Japan), best-corrected visual acuity (BCVA), slit-lamp biomicroscopy, intraocular pressure (Full Auto Tonometer TX-F; Topcon, Japan), intraocular lens master (Carl Zeiss Meditec, Germany). Swept-source optical coherence tomography (SS-OCT, DRI OCT-1 Atlantis; Topcon, Japan) was used in a mode of 12 radial scans with scan length of 9 mm. Fundus photography centered on the macula was obtained using the same SS-OCT, which was equipped with a digital, nonmydriatic retinal camera. The value of image quality (IQ) was calculated automatically by the embedded software on SS-OCT. The image was defined as low quality, when the IQ value was less than 60. The spherical equivalent refraction (SER) was obtained as spherical degree plus half of cylindrical degree. The BCVA was converted to the logarithm of minimal angle of resolution (logMAR).

### Myopic maculopathy classification

ATN classification was used for grading myopic maculopathy. This system contained three important myopic maculopathy including atrophy (A), traction (T), and neovascularization (N). Because all study eyes had FD, we only evaluated the A and N component. There are five categories in A component: no myopic retinal lesions (A0); tessellated fundus only (A1); diffuse chorioretinal atrophy (A2); patchy chorioretinal atrophy (A3); and complete macular atrophy (A4). There are four categories in N component: no myopic choroidal neovascularization (N0); macular lacquer cracks (N1); active choroidal neovascularization (N2a); scar or Fuch’s spot (N2s). The grading of myopic maculopathy was performed by two independent, well-trained graders (J.Y.F. and J.Y.Y.). In cases of disagreement, a retinal specialist (Y.F.) would make a judgement. The agreement between two graders was excellent for atrophic maculopathy (weighted kappa = 0.784, *P* < 0.001) and neovascular maculopathy (weighted kappa = 0.802, *P* < 0.001).

### Surgical procedure

A standard 3-port 23-gauge pars plana vitrectomy was performed using a Constellation device (Alcon, Fort Worth, TX) by the same surgeon (Y.F.). After core vitrectomy, triamcinolone acetonide (TA, 2.5 mg/ml) was applied in assistance of removing the posterior hyaloid. Then peripheral vitreous was removed by a complete vitrectomy. The ILM was stained by indocyanine green solution (1.5 mg/ml) and peeled using intraocular forceps around the fovea to a region of approximately three optic disk diameters. Complete fluid-air exchange was conducted using sterilized air. The application of silicon oil was according to the surgeon’s judgement.

### Statistical analysis

All statistical analyses were performed by SPSS software for Windows (version 21.0; IBM Corp., Armonk, NY, USA). The Mann-Whitney test or a t-test was used to analyze the differences between two groups as appropriate. Spearman correlation analysis was used to detect the associations between the grade of myopic maculopathy and baseline parameters in FD eyes. Linear regression analysis was performed to investigate the factors influencing postoperative BCVA and the retinal reattachment period. A logistic regression model was applied to assess the factors influencing the occurrence of postoperative secondary macular holes. A *P* value < 0.05 was considered statistically significant.

## Results

 We retrospectively reviewed the records of 61 FD eyes from 59 pathological myopia patients (18 males and 43 females). Baseline demographic information is presented in Table 1. The average age of the patients was 59.97 ± 9.63 years (range: 38–83 years old). The mean axial length and SER were 29.57 ± 1.69 mm (range: 26.03–33.22 mm) and − 13.56 ± 4.18 D (range: -6.00 - -24.00D). The mean duration of symptoms and follow-up were 4.28 ± 4.77 months and 30.9 ± 6.46 months, respectively. At the baseline visits, 52 eyes (85.2%) had posterior staphyloma and 9 eyes (14.8%) were pseudophakic.

**Table 1:** Baseline characteristics of the study population

Baseline parameters	FD eyes (n = 61)
**Age (years)**	
Mean ± SD	59.97 ± 9.63
Range	38–83
**Gender**	
Male, n (%)	18 (29.5%)
Female, n (%)	43 (70.5%)
**Duration of symptoms (months)**	
Mean ± SD	4.28 ± 4.77
Range	0.5–24
**SER (D)**	
Mean ± SD	-13.56 ± 4.18
Range	-6.00 - -24.00
**Axial length (mm)**	
Mean ± SD	29.57 ± 1.69
Range	26.03–33.22
**Baseline BCVA (logMAR)**	
Mean ± SD	1.34 ± 0.52
**Posterior staphyloma, n (%)**	52 (85.2%)
**Lens status**	
Pseudophakic, n (%)	9 (14.8%)
**Baseline macular complications**	
Macular retinoschisis, n (%)	57 (93.4%)
Vitreomacular traction, n (%)	17 (27.9%)
Outer lamellar macular hole, n (%)	27 (44.3%)
Inner lamellar macular hole, n (%)	10 (16.4%)

*FD *foveal detachment, *SER *spherical equivalent refraction, *BCVA *best corrected visual acuity, *logMAR *logarithm of the minimum angle of resolution

**Fig. 1:**
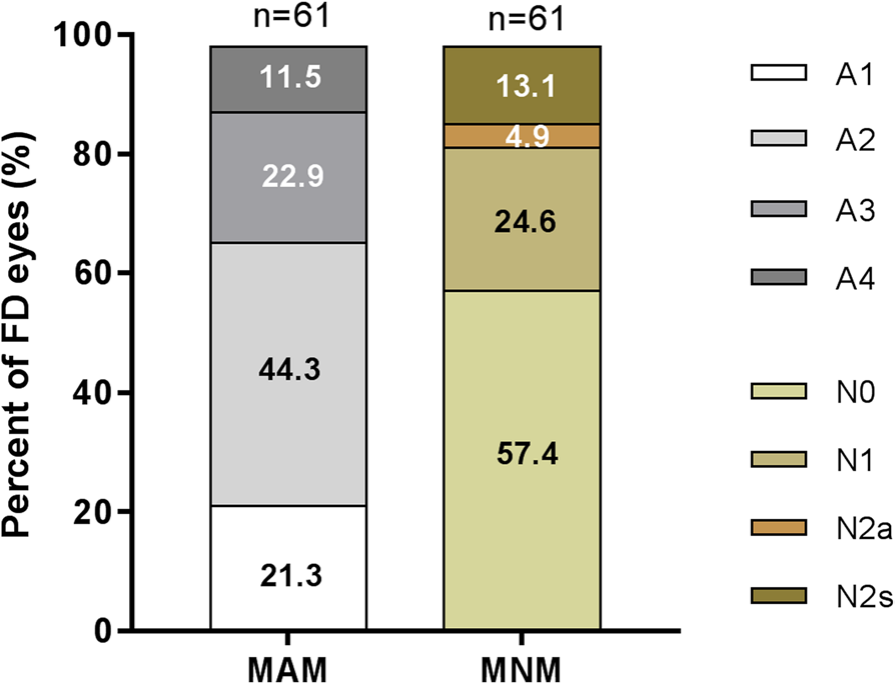
Distributions of MAM and MNM grade in FD eyes at baseline. All FD eyes had MAM. A2 was observed in 27 eyes (44.3%), followed by A3, A1 and A4 which were observed in 14 eyes (22.9%), 13 eyes (21.3%) and 7 eyes (11.5%) respectively. Over half of FD eyes (57.4%) didn’t has MNM (N0). N1, N2a and N2s were detected in 15 eyes (24.6%), 3 eyes (4.9%) and 8 eyes (13.1%) respectively

**Fig. 2:**
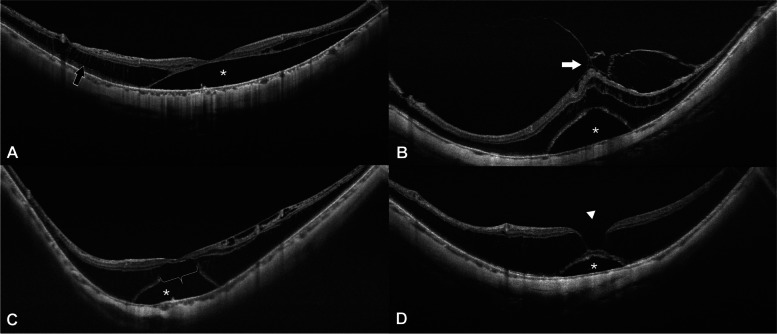
Representative OCT images of four kinds of complication accompanied by FD at baseline. (**A**) FD (asterisk) accompanied by macular retinoschisis (black arrows). (**B**) FD accompanied by vitreomacular traction (white arrows). (**C**) FD accompanied by outer lamellar macular hole (white brace). (**D**) FD accompanied by inner lamellar macular hole (white arrowhead)

The morphological characteristics of the macula in FD eyes were evaluated according to the ATN classification. All FD eyes had myopic atrophy maculopathy (MAM). The most common category was A2, which was observed in 27 eyes (44.3%). A3, A1 and A4 were observed in 14 eyes (22.9%), 13 eyes (21.3%) and 7 eyes (11.5%), respectively. The prevalence of myopic neovascular maculopathy (MNM) in FD eyes was 42.6% (26 eyes). Among these, 15 eyes had N1 (24.6%). N2a and N2s were detected in 3 eyes (4.9%) and 8 eyes (13.1%), respectively (Fig. 1). In addition to MAM and MNM, we observed four macular lesions accompanied by FD at baseline (Fig. 2). The most prevalent complication was macular retinoschisis, which was detected in 57 eyes (93.4%). Vitreomacular traction (VMT) was observed in 17 eyes (27.9%). Outer lamellar macular hole (O-LMH) and inner lamellar macular hole (I-LMH) were observed in 27 eyes (44.3%) and 10 eyes (16.4%), respectively. Correlation analysis found that higher MAM grade was accompanied by higher MNM grade in FD eyes (*r* = 0.662, *P* = 0.000). The results also showed that FD patients of older age (*r* = 0.262, *P* = 0.042), female gender (*r* = -0.282, *P* = 0.027), higher SER (*r* = -0.260, *P* = 0.043), and longer axial length (*r* = 0.339, *P* = 0.008) tended to have more severe MAM. The MNM grade revealed a negative association with the occurrence of O-LMH (*r* = -0.305, *P* = 0.017). FD eyes with more severe MAM (*r* = 0.422, *P* = 0.001) or MNM (*r* = 0.402, *P* = 0.001) were likely to have worse baseline logMAR BCVA (Table 2).

**Table 2:** Correlations between MAM or MNM grade and baseline parameters in FD eyes

Baseline parameters	MAM grade		MNM grade	
	*r*	*P* value	*r*	*P* value
**Age (years)**	0.262	**0.042**	0.262	**0.041**
**Gender**	-0.282	**0.027**	-0.229	0.076
**Duration of symptoms (m)**	0.052	0.693	0.135	0.305
**SER (D)**	-0.260	**0.043**	-0.108	0.408
**Axial length (mm)**	0.385	**0.002**	0.140	0.282
**Baseline BCVA (logMAR)**	0.422	**0.001**	0.402	**0.001**
**MAM grade**			0.662	**< 0.001**
**MNM grade**	0.662	**< 0.001**		
**Baseline macular complications**				
Macular retinoschisis	-0.100	0.445	0.053	0.686
Vitreomacular traction	0.014	0.913	0.040	0.762
Outer lamellar macular hole	-0.152	0.241	-0.305	**0.017**
Inner lamellar macular hole	0.008	0.951	-0.018	0.888

*MAM *myopic atrophy maculopathy, *MNM *myopic neovascular maculopathy, *SER *spherical equivalent refraction, *BCVA* best corrected visual acuity, *logMAR* logarithm of the minimum angle of resolution

*P* value was based on Spearman’s correlation test, significant difference bolded

**Fig. 3:**
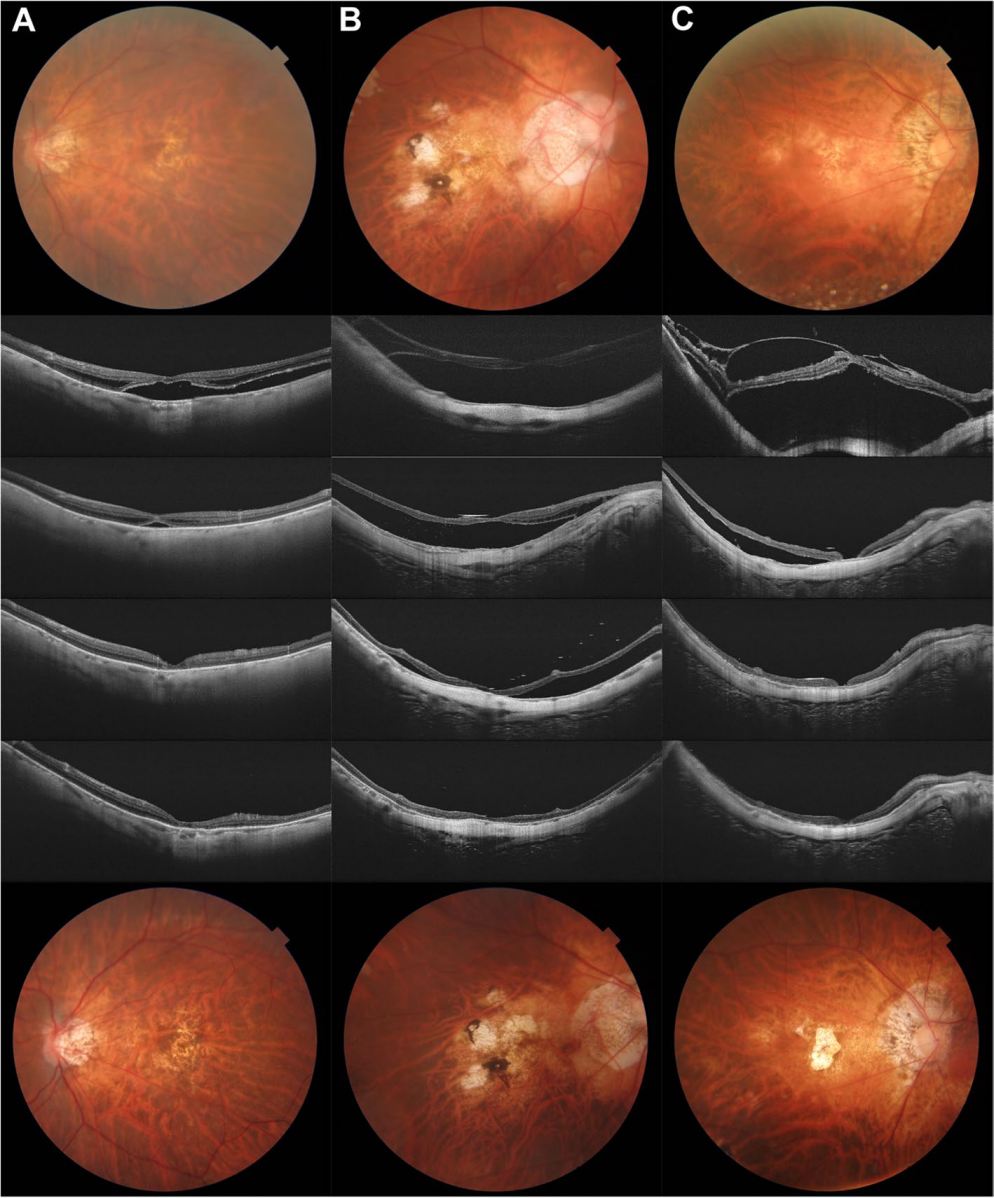
Representative images of FD eyes. (First row) baseline fundus photographs. (Second row) baseline SS-OCT images. (Third row) SS-OCT images at 6 months after surgery. (Forth row) SS-OCT images at 12 months after surgery. (Fifth row) SS-OCT images at 24 months after surgery. (Sixth row) fundus photographs at 24 months after surgery. (**A**) Left eye of a 63-year-old female patient with tessellated fundus and without neovascular change (A1T3N0) underwent vitrectomy with air tamponade. (**B**) Right eye of a 69-year-old female patient with patchy chorioretinal atrophic fundus and Fuchs spot in the macular area (A3T3N3) underwent vitrectomy and oil tamponade. (**C**) Right eye of a 68-year-old male patient with diffuse chorioretinal atrophic fundus (A2T3N0), accompanied by VMT and O-LMH, underwent vitrectomy and air tamponade. The patient had secondary macula hole after primary surgery and accepted a secondary vitrectomy and oil tamponade

Next, we investigated the anatomical and functional outcomes of vitrectomy in FD eyes. At the end of the follow-up period, FD had completely resolved in 59 of 61 eyes (96.7%). Two eyes had residual subretinal fluid. The mean duration of retinal reattachment was 12.10 ± 8.10 months (range: 0.5–34 months). BCVA improved from 1.34 ± 0.52 preoperatively to 0.83 ± 0.43 logMAR at 24 months postoperatively (*t* = 8.204, *P* < 0.001). In the postoperative period, 8 of 61 eyes (13.1%) had secondary macular holes. The mean time for the occurrence of secondary macular hole was 3.4 ± 4.1 weeks after primary vitrectomy, ranging from 1 week to 10 weeks. After undergoing reoperation with vitrectomy and silicone oil tamponade, all 8 eyes had achieved macular hole closure and retinal reattachment at the end of follow-up. Representative cases are shown in Fig. 3.

**Fig. 4:**
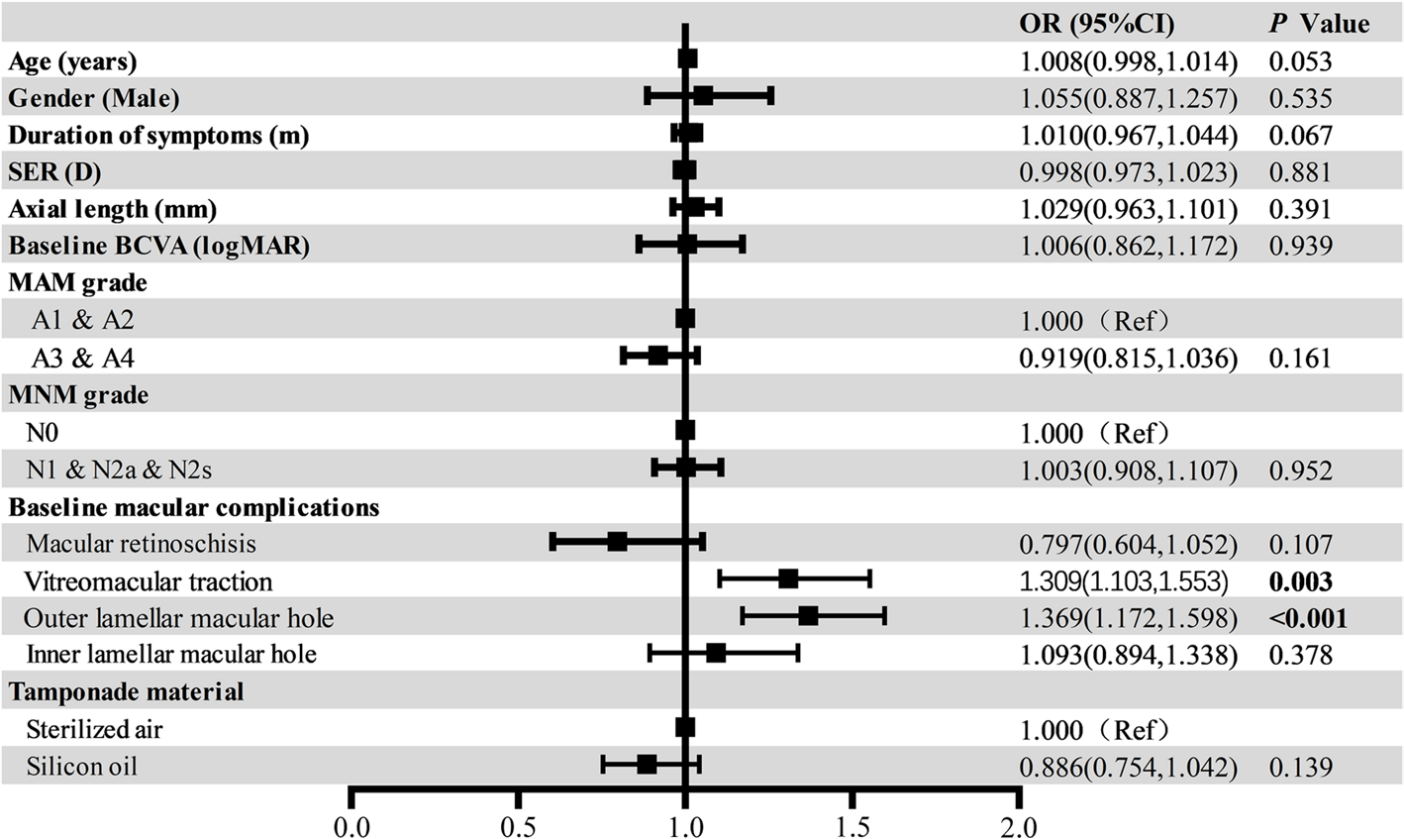
Associations between preoperative ocular parameters and the occurrence of secondary macular hole after vitrectomy for FD in the logistic regression model. After adjustment of age and gender, FD eyes accompanied by VMT (OR = 1.309, *P* = 0.003) or O-LMH (OR = 1.369, *P* < 0.001) at baseline were more likely to develop secondary macular hole after vitrectomy. No significant associations were found between occurrence of secondary macular hole and duration of symptoms, SER, axial length, preoperative BCVA, MAM grade, MNM grade or tamponade material (*P* > 0.05)

Potential factors influencing postoperative BCVA, retinal reattachment period and secondary macular hole development in FD eyes were analyzed by a linear or logistic regression model after adjustment for age and gender. No significant associations were found between surgical outcomes and age, axial length, SER or duration of symptoms (*P* > 0.05). Baseline BCVA (*B* = 0.354, *P* < 0.001) was a positive prognostic factor for postoperative BCVA, while baseline MAM grade (*B* = 0.213, *P* = 0.005) and VMT (*B* = 0.292, *P* = 0.007) were adverse prognostic factors for postoperative BCVA. Interestingly, FD eyes with macular retinoschisis at baseline tended to have better postoperative BCVA (*B* = -0.539, *P* = 0.002). In addition, our results showed that the duration of retinal reattachment was positively associated with baseline MAM grade (*B* = 5.670, *P* = 0.002) but negatively associated with MNM grade (*B* = -3.503, *P* = 0.020) (Table 3). FD eyes with VMT (OR = 1.309, *P* = 0.003) or O-LMH (OR = 1.369, *P* < 0.001) at baseline were more likely to develop secondary macular hole after vitrectomy (Fig. 4).

**Table 3:** Linear regression analysis of postoperative BCVA or foveal reattachment period and baseline parameters in the model

Baseline parameters	Postoperative BCVA (logMAR)				Foveal reattachment period (m)			
	*B*	*Se (B)*	*P* value	95% CI	*B*	*Se (B)*	*P* value	95% CI
**Age (years)**	-0.004	0.005	0.470	-0.013 to 0.006	-0.011	0.120	0.928	-0.253 to 0.231
**Gender**	0.114	0.104	0.281	-0.096 to 0.323	1.848	2.564	0.475	-3.313 to 7.009
**Duration of symptoms (m)**	0.009	0.009	0.299	-0.009 to 0.027	-0.256	0.219	0.249	-0.696 to 0.185
**SER (D)**	-0.010	0.015	0.520	-0.040 to 0.020	-0.088	0.367	0.812	-0.827 to 0.652
**Axial length (mm)**	-0.058	0.040	0.154	-0.138 to 0.023	-1.757	0.983	0.080	-3.736 to 0.221
**Baseline BCVA (logMAR)**	0.354	0.092	**< 0.001**	0.170 to 0.539	0.464	2.258	0.838	-4.082 to 5.009
**MAM grade**	0.213	0.072	**0.005**	0.069 to 0.358	5.670	1.763	**0.002**	2.121 to 9.220
**MNM grade**	-0.013	0.059	0.826	-0.132 to 0.106	-3.503	1.458	**0.020**	-6.438 to -0.568
**Baseline macular omplications**								
Macular retinoschisis	-0.539	0.166	**0.002**	-0.873 to -0.204	-2.475	4.087	0.548	-10.702 to 5.752
Vitreomacular traction	0.292	0.102	**0.007**	0.085 to 0.498	0.406	2.521	0.873	-4.668 to 5.481
Outer lamellar macular hole	-0.025	0.093	0.785	-0.212 to 0.161	-2.261	2.279	0.326	-6.850 to 2.327
Inner lamellar macular hole	0.085	0.121	0.482	-0.157 to 0.328	-0.366	2.965	0.902	-6.335 to 5.602
**Tamponade material**	-0.188	0.097	0.058	-0.383 to 0.007	1.027	2.380	0.668	-3.764 to 5.819

Se (B) = standard error of B coefficient, SER = spherical equivalent refraction, BCVA = best corrected visual acuity, logMAR = logarithm of the minimum angle of resolution, MAM = myopic atrophy maculopathy, MNM = myopic neovascular maculopathy

*P* value was based on linear regression model, significant difference bolded

Adjusted R^2^ for postoperative BCVA = 0.465, for foveal reattachment period = 0.525

## Discussion

This research is, to the best of our knowledge, the first to apply the ATN classification to evaluate long-term surgical outcomes and prognostic factors in FD eyes. Our results showed the following: (1) Vitrectomy with ILM peeling was effective in the long-term for treating FD. (2) FD eyes with VMT or O-LMH were more likely to develop secondary macular hole after vitrectomy. (3) FD eyes with more severe MAM tend to have poorer postoperative BCVA and extended periods of retinal reattachment.

With the development of OCT, MTM including retinoschisis, foveal detachment, macular hole and macular hole retinal detachment has attracted more attention. However, clinical intervention for MTM is complex and still controversial. According to the natural course of MTM, myopic macular retinoschisis appears first at the early stage, and in severe cases, will evolve to FD and cause vision loss [14]. However, considering the variable natural prognosis of myopic retinoschisis, using surgical approaches for all retinoschisis may not be the optimal choice for MTM management. In this study, we observed that 93.44% of the FD eyes had macular retinoschisis, and nearly half had outer or inner laminar holes. The rupture of the outer retina in FD eyes is thought to render the eyes prone to developing full-thickness macular holes [7, 15]. Our observation supported the view that FD is an intermediate stage in the development of MTM. In eyes with retinoschisis, nutrient diffusion from the choriocapillaris to the photoreceptors might not be disturbed. Once the fovea detaches, photoreceptor damage progresses, and therefore vision decreases. Thus, we deduced that the presence of FD is a sign of MTM exacerbation and that surgical intervention for FD may be a suitable option for MTM management.

Vitrectomy is the most popular surgical procedure for MTM including retinoschisis and macular hole [16, 17]. However, few studies have focused on the surgical management of FD. In our study, we achieved complete resolution of FD in 96.7% of eyes, which was a higher rate of resolution than in other studies in which it was reported to be between 70 and 88.9% [18, 19]. This may be related to the long follow-up period of our study. Our results revealed that the mean duration of retinal reattachment reached 12.10 ± 8.10 months, with the longest reattachment duration being 34 months. This indicates that a relatively short-term follow-up period may omit reattached cases and produce observational bias. Secondary macular hole was the major postoperative complication, with an incidence of 13.1% (8/61). Most secondary macular holes developed in eyes with VMT and O-LMH at baseline and usually occurred within 1 week after surgery. It is thought that although ILM peeling ensures the complete removal of premacular vitreous traction and decreases retinal rigidity, it may be a risk factor for the development of macular holes, especially in eyes with a very thin or excessively stretched fovea. Recently, a fovea sparing ILM peeling technique was introduced for MTM [20, 21]. This modified technique had a rate of postoperative macular hole development of 1.96% in a report for retinoschisis with FD, which was much lower than that of the traditional ILM peeling technique [20]. None of the eyes in our cases had developed a late occurrence of macular holes or retinal detachment at the last follow-up, which implies that vitrectomy is effective for blocking the progression of FD to more severe MTM.

Thus far, the preoperative evaluation of MTM has mainly focused on tractional lesions. However, many MTM patients also show atrophic or neovascular features of pathologic myopia. A single evaluation on tractional lesion may ignore the effect of other myopic maculopathies on the prognosis of MTM surgery. In 2019, Jorge Ruiz-Medrano et al. proposed a new grading system known as the ATN classification system [12]. The ATN criteria integrate the atrophic, tractional and neovascular components of the existing grading system. Thus, we applied the ATN classification to comprehensively evaluate the prognostic factors for FD surgery. In our study, all FD eyes had different grades of MAM. FD eyes with more severe MAM were likely to have a worse baseline and postoperative BCVA. Fundamentally, the main pathology of atrophic maculopathy is thinning of the choroid and retina [21]. As MAM grade increases, atrophy progresses and results in the loss of outer retina, including the RPE, photoreceptors and retinal nerve fiber layer, which have a greater influence on the potential for visual function recovery [22, 23]. In addition, we found that the duration of retinal reattachment was positively associated with baseline MAM grade. It is hypothesized that choroidal circulation is reduced in atrophic lesions due to the loss of choriocapillaris or occlusion of large choroidal vessels [24]. The change in choroidal hemodynamics accompanied by RPE loss and stretching of the retina causes reduced absorption of sub-foveal fluid. We found a correlation between the severity of MAM and MNM in FD eyes. However, the MNM grade had no correlation with preor postoperative BCVA. This may be because most FD eyes with MNM in our study were at the N1 stage (lacquer cracks). At this primary stage, neovascularization develops slowly and does not cause long-term vision loss [25]. Choroidal atrophy developing around the regressed neovascular lesion, secondary to RPE damage, likely plays a key role in the visual prognosis of FD eyes.

Several potential limitations exist in this study. First, due to the retrospective observational nature, the postoperative follow-up time points had difference among patients, which may produce bias. Second, considering the posterior staphyloma is an important characteristic of MTM, we lack of 3D-MRI image to accurately identify ocular shape. Third, as modified surgical techniques have been applied for MTM such as fovea sparing ILM peeling and posterior scleral reinforcement, further investigation on the comparison of different techniques is needed.

## Conclusions

In summary, careful consideration is necessary for FD eyes with accompanying VMT or O-LMH because of a high risk for developing secondary macular hole after vitrectomy. In addition, a more severe preoperative MAM indicates a worse BCVA recovery and longer retinal reattachment. Thus, it is important to assess the MAM category when considering surgical management and evaluating the prognosis of FD eyes.

## Data Availability

The datasets created and/or analyzed during the current study available from the corresponding author upon reasonable request.
